# Abnormal Static and Dynamic Functional Network Connectivity in Patients With Presbycusis

**DOI:** 10.3389/fnagi.2021.774901

**Published:** 2022-01-05

**Authors:** Chunhua Xing, Yu-Chen Chen, Song’an Shang, Jin-Jing Xu, Huiyou Chen, Xindao Yin, Yuanqing Wu, Jin-Xia Zheng

**Affiliations:** ^1^Department of Radiology, Nanjing First Hospital, Nanjing Medical University, Nanjing, China; ^2^Department of Otolaryngology, Nanjing First Hospital, Nanjing Medical University, Nanjing, China; ^3^Department of Radiology, Nanjing Maternity and Child Health Care Hospital, Women’s Hospital of Nanjing Medical University, Nanjing, China

**Keywords:** presbycusis, static functional network connectivity, cognitive impairment, dynamic functional network connectivity, functional magnetic resonance imaging

## Abstract

**Aim:** This study aimed to investigate abnormal static and dynamic functional network connectivity (FNC) and its association with cognitive function in patients with presbycusis.

**Methods:** In total, 60 patients with presbycusis and 60 age-, sex-, and education-matched healthy controls (HCs) underwent resting-state functional MRI (rs-fMRI) and cognitive assessments. Group independent component analysis (ICA) was carried out on the rs-fMRI data, and eight resting-state networks (RSNs) were identified. Static and dynamic FNCs (sFNC and dFNC) were then constructed to evaluate differences in RSN connectivity between the patients with presbycusis and the HCs. Furthermore, the correlations between these differences and cognitive scores were analyzed.

**Results:** Patients with presbycusis had differences in sFNC compared with HCs, mainly reflected in decreased sFNC in the default mode network (DMN)-left frontoparietal network (LFPN) and attention network (AN)-cerebellum network (CN) pairs, but they had increased sFNC in the auditory network (AUN) between DMN domains. The decreased sFNC in the DMN-LFPN pair was negatively correlated with their TMT-B score (*r* = –0.441, *p* = 0.002). Patients with presbycusis exhibited aberrant dFNCs in State 2 and decreased dFNCs between the CN and AN and the visual network (VN). Moreover, the presbycusis group had a shorter mean dwell time (MDT) and fraction time (FT) in State 3 (*p* = 0.0027; *p* = 0.0031, respectively).

**Conclusion:** This study highlighted differences in static and dynamic functional connectivity in patients with presbycusis and suggested that FNC may serve as an important biomarker of cognitive performance since abnormal alterations can better track cognitive impairment in presbycusis.

## Introduction

Presbycusis (or age-related hearing loss) is the most pervasive sensory deficit affecting elderly individuals and manifests with progressive, bilateral, sensorineural high-frequency hearing loss (Gates and Mills, 2005), resulting from the cumulative influence of aging on the auditory nervous system and binaural hearing ability (Uchida et al., 2019). This disorder is mainly characterized by reduced auditory sensitivity, impaired sound perception and localization, and decreased ability to distinguish speech in noisy environments (Gates and Mills, 2005) and has become the third most prevalent health disorder affecting elderly individuals, after heart disease and arthritis (Mahmoudi et al., 2021). Numerous studies have demonstrated that presbycusis is strongly associated with cognitive decline and impairment to cognitive-related domains, which may precipitate the early landmarks of dementia (Ford et al., 2018; Loughrey et al., 2018). Given that hearing loss is the most modifiable risk factor for cognitive impairment (Slade et al., 2020), early detection and intervention are particularly important. Thus, elucidating the underlying neural mechanisms between hearing loss, related neurological changes, and cognitive function is of great significance.

Resting-state functional MRI (rs-fMRI), a prominent non-invasive tool for the localization and lateralization of brain functions, has gradually become the focus of neuroscience research in recent years (Smitha et al., 2017). “Resting-state” refers to the absence of any stimulus or task during the fMRI analysis, while functional connectivity (FC) can reflect significant temporal correlations between spontaneous neurophysiological events in spatially remote brain areas (Biswal et al., 1995). At present, a large number of studies have employed rs-FC to detect the functional organization of the brain in presbycusis. Previous fMRI studies have found that age-related hearing loss contributes to reduced activation in central auditory pathways, dysfunctional connectivity between auditory and limbic networks (Rutherford et al., 2018), and decreased directed FC between the middle temporal gyrus (MTG), insula, and hippocampus (Chen et al., 2020). While the compensatory activation of the frontal lobe and cognitive control network increased, hearing-impaired individuals also compensated for their difficulty in understanding speech through supplemental executive function, thus leading to cascading cognitive effects that further affected cognitive processing, such as perception, comprehension, and working memory (Peelle and Wingfield, 2016; Loughrey et al., 2018). Similarly, earlier hearing loss may cause an increase in the FC of networks, such as the visual and sensorimotor networks (VN and SMN), indicating that cross-modal plastic reorganization can occur after loss of function in the auditory-deprived brain (Schulte et al., 2020). However, resting-state functional coupling changes with the progression of presbycusis, as the aggravating degree of hearing loss is accompanied by increased audiovisual integration and decreased connectivity between AUN and motor-related network (Schulte et al., 2020).

It is worth noting that the abovementioned studies adopted the traditional rs-FC approach, which assumes that the functional interactions are spatially and temporally stationary. However, functional connections across networks are increasingly considered to be dynamic, i.e., they fluctuate over time (Meier et al., 2016). These time-varying characteristics represent transient and recurring whole-brain patterns of temporal coupling, which reveal neural mechanisms that cannot be detected by static rs-FC. Therefore, an analytical framework based on the group independent component analysis (ICA) (Olivito et al., 2020), the sliding window approach, and the k-means clustering was proposed to characterize the dynamic changes in disorder-related whole-brain rs-FC or functional network connectivity (FNC) (Fu et al., 2018, 2019). Previous studies have applied this approach to diseases such as autism (Fu et al., 2019), Parkinson’s disease (Kim et al., 2017), and schizophrenia (Fu et al., 2018), confirming that dynamic FNC (dFNC) serves as a useful biomarker and showing that more details can be obtained through dFNC than otherwise accessible through the static connectivity analyses of neurodegenerative diseases.

In our previous study, significant decreases in static FNC (sFNC) between the auditory network (AUN) and the default mode network (DMN) were correlated with cognitive scores, suggesting that sFNC may be used to evaluate the onset and progression of cognitive impairment in presbycusis and to provide potential clues for clinical treatment (Xing et al., 2020). No prior studies have investigated changes in sFNC and dFNC in presbycusis individuals. Thus, we hypothesized that the sFNC, dFNC, and temporal characteristics of dynamic FC states might be able to describe the underlying nature of cognitive impairment in presbycusis more accurately. To this end, we combined dFNC with sFNC approaches to comprehensively explore the changes in whole-brain connectivity patterns from both static and dynamic aspects and to reveal the relationship between altered FNC features and patterns and cognitive impairment.

## Materials and Methods

### Participants

All 60 patients with presbycusis were recruited from the Department of Otolaryngology in Nanjing First Hospital, and 60 healthy controls (HCs), who were age-, sex-, education-, and handedness-matched, were recruited *via* the community health census or advertisements. Approval for the study was obtained from the Research Ethics Committee of Nanjing Medical University, and each individual provided written informed consent before their participation in this study. Pure-tone audiometry was carried out by a clinical audiometer to measure the pure tone average (PTA) and to determine the hearing level of individuals using seven different octave frequencies (i.e., 0.125, 0.25, 0.5, 1, 2, 4, and 8 kHz). For the presbycusis group, the inclusion criterion of the audiometric threshold was a PTA > 25 dB HL in the better hearing ear; for the HCs, normal hearing was defined as a PTA < 25 dB HL for all 7 frequencies. Moreover, to confirm normal functioning of the middle ear, tympanometry was measured using a Madsen Electronics Zodiac 901 Middle Ear Analyzer (GN Otowrics).

Nine participants in the presbycusis group were excluded from the study due to excessive head movement during MRI scans. In addition, to obtain a more homogenous sample, the study excluded individuals who suffered from ear diseases other than presbycusis that affected hearing thresholds and sensorineural hearing loss, including tinnitus (Baguley et al., 2013), hyperacusis (Khalfa et al., 2002), Meniere’s disease, and otosclerosis, as well as individuals with a previous history of occupational noise exposure (Guest et al., 2017), otologic surgery, ototoxic drug therapy, or hearing aid use. Individuals who were addicted to smoking and alcoholism had depression, brain damage, epilepsy, major illnesses (i.e., anemia, thyroid dysfunction, and cancer), or mental or neurological disorders, as well as individuals with contraindications to MRI were also excluded.

### Cognitive Assessment

We conducted a series of cognitive assessments in a fixed order to reveal various aspects of cognitive functioning, mainly focusing on memory, attention, and executive functions. The tests conducted on all participants were as follows: the Mini-Mental State Exam (MMSE) (Ardila et al., 2016) and the Montreal Cognitive Assessment (MoCA) (Dawes et al., 2019) to assess general cognitive function including verbal abilities and cognitive reserve; the Auditory Verbal Learning Test (AVLT) (Xu et al., 2020) and Complex Figure Test (CFT) (Shin et al., 2006) to investigate episodic verbal learning as well as visual memory recall; and the Digit Span Test (DST) (Gabel et al., 2019) to measure working memory. Additionally, attentiveness and executive functions were assessed by the Trail Making Test (TMT) (Sanchez-Cubillo et al., 2009) and Clock-Drawing Test (CDT) (Viscogliosi et al., 2017). The TMT consists of two subtests: TMT-A mainly assesses motor speed and visual search skills, while TMT-B reflects cognitive flexibility. Furthermore, the Digit Symbol Substitution Test (DSST) (Rosano et al., 2013) and Verbal Fluency Test (VFT) (Vaucheret Paz et al., 2020) were used to measure both the mental operating speed and visuospatial abilities. Additionally, the symptoms of anxiety and depression were evaluated by the hospital anxiety and depression scale (Pais-Ribeiro et al., 2018). In total, it took approximately 60 min for each individual to complete all these tests.

### Imaging Data Acquisition and Preprocessing

A 3.0 Tesla Philips MRI scanner (Ingenia, Netherlands) with an eight-channel phased-array head coil was used to collect imaging data from all participants. During the data acquisition, participants were instructed to lie quietly, keep still, and keep their eyes closed but not to fall asleep or think about anything special. Foam padding and paper tape were used to reduce involuntary head movements, and earplugs were used to decrease the influence of noise. According to the specifications of the manufacturer, the earplugs (Hearos Ultimate Softness Series, Buffalo, NY, United States) could attenuate scanner noise by almost 32 dB. All the participants completed an 8-min and 8-s rs-fMRI scan with a gradient echo-planar imaging (EPI) sequence: repetition time (TR) = 2,000 ms, echo time (TE) = 30 ms, slices = 36, thickness = 4 mm, gap = 0 mm, matrix size = 64 × 64, field of view (FOV) = 240 mm × 240 mm, and flip angle (FA) = 90° with voxel size = 3.75 mm × 3.75 mm × 4.0 mm. The structural images were acquired using a three-dimensional turbo fast echo (3D-TFE) T1-weighted (T1WI) sequence with an FOV = 256 mm × 256 mm; matrix size = 256 × 256; TR/TE = 8.1/3.7 ms, slices = 170, thickness = 1 mm, gap = 0 mm, and FA = 8°, which lasted for 5 min and 29 s. Furthermore, all scans were obtained with parallel imaging using the sensitivity encoding (SENSE) technique and the SENSE factor = 2.

The functional image data were preprocessed by the Graph Theoretical Network Analysis (GRETNA) to make the data suitable for further analysis. The specific steps were as follows: first, the first 10 volumes were deleted to reach a steady-state of magnetization and to allow participants to adapt to the scanning environment. Second, default slice timing routines in GRETNA were used to correct differences in image acquisition time between slices, with the middle slice serving as the reference slice. Third, the functional data were realigned to correct for head motion (realignment), and any subjects with a head motion greater than 2.0-mm translation or 2.0 rotation in any direction were excluded. Other preprocessing steps included spatial normalizing of corrected volumes to the Montreal Neurological Institute space by EPI template with resampled voxel size = 3 mm × 3 mm × 3 mm and spatial smoothing with a 6-mm full width at half maximum Gaussian smooth kernel.

### Group Independent Component Analysis and Resting-State Networks Identification

We used the group ICA function of the fMRI Toolbox (GIFT) to implement the spatial group ICA and to detect the resting-state networks (RSNs), which involved the following steps: first, the principal component analysis (PCA) was conducted to reduce the data dimensionality. The number of ICs for all participants was automatically estimated using the minimum description length (MDL) criteria (resulting in 34 ICs), and another PCA was performed to achieve the remaining dimensionality reduction. Second, the InfoMax algorithm was used to run the appropriate ICA. Finally, the time courses and spatial maps of individual subjects were back-reconstructed by group ICA (Wang et al., 2020), and the results were transformed to *Z*-scores for display. Ten significant components were identified as RSNs through visual observation of the ICA results based on previous studies. These ICs were classified into eight RSNs, namely, AUN, DMN, attention network (AN), left frontoparietal network (LFPN), right frontoparietal network (RFPN), SMN, VN, and cerebellum network (CN), all of which have been widely reported in previous rs-fMRI research (Jiang et al., 2020; Wang et al., 2020).

### Static Functional Network Connectivity

After ICA analysis, the MANCOVAN toolbox in GIFT was used to calculate the correlations between any two RSN time courses for each individual. Then, the FNC (temporal correlation) was acquired by computing the Pearson’s correlation coefficient between each summary time course and every other summary time course, thus generating a 10 × 10 matrix for each participant. The correlation results were Fisher *Z*-transformed before further analysis. Finally, we used a general linear model (GLM) with age, sex, and education as nuisance covariates to determine which pair of RSNs was significantly different between the groups. The significance threshold was set at *p* < 0.05, and multiple comparisons were corrected using false discovery rate (FDR).

### Dynamic Functional Network Connectivity

The temporal dFNC module, as implemented in the GIFT software package, was used to perform the dFNC analysis. Preprocessing included cubic detrending, despiking using 3D-despike, and low-pass filtering using a high-frequency cutoff of 0.15 Hz to decrease the impact of artifacts (Wang et al., 2020). Then, a sliding time-window approach [window size set to 15 TRs of a rectangular window convolved with a Gaussian (= 3 TRs)] was employed to compute the dFNC between ICA time courses (Du et al., 2016). Thus, a total of 21,090 windowed FNC (wFNC) symmetric matrices (111 participants with 190 wFNCs) were constructed. Then, the k-means clustering algorithm (using the squared Euclidean distance method with 500 iterations and 150 replicate dFNC windows) was conducted on the wFNC matrices (Malhi et al., 2019). The most commonly used method to determine the optimal value of *k*, which is defined as the ratio of within- to between-cluster distances, is the k-means clustering algorithm based on the elbow criterion; the goal of the algorithm is to minimize *k*. The turning point (or elbow) of our results was at 4 FNC states, which reflects the optimal number of clusters (Jiang et al., 2020). Two-sample *t*-tests were used to compare each of the 45 mean dFNC correlations (10 × 9/2) from each of the 4 states between groups, with a significance threshold of *p* < 0.05 (FDR-corrected). In addition, three dFNC indices were extracted from all four states of each subject (Jiang et al., 2020), namely, fraction time (FT) of each state, mean dwell time (MDT), and number of transitions (NT). FT indicates the percentage of time spent in each state out of the total time, MDT reflects the average length of time the subjects spent in a certain state, and NT refers to the number of times a subject switched between different states. These indices were compared by the non-parametric Mann–Whitney *U*-tests; *p* < 0.05 was considered statistically significant.

### Statistical Analyses

The IBM SPSS 19.0 software package was used to investigate the differences in demographic and clinical information between the patients with presbycusis and the HC groups. Chi-square tests were used for categorical variables, and the independent two-sample *t*-tests were used for continuous variables. Additionally, significant differences between the two groups were explored for the eight RSNs, and static and dFNC coefficients were used to calculate correlations with cognitive assessment scores *via* Pearson’s or Spearman’s correlation analysis. The dFNC values, such as FT, MDT, and NT, were also analyzed for correlation with cognitive assessment scores; *p* < 0.05 was considered statistically significant.

## Results

### Demographic Data and Cognitive Status

**FIGURE 1:**
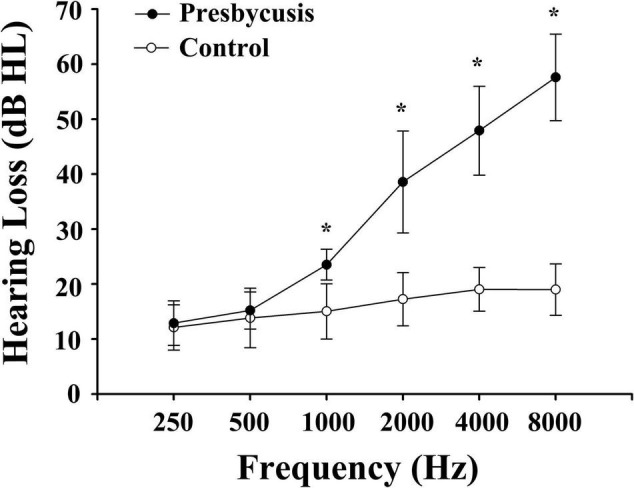
Average hearing thresholds of patients with presbycusis and healthy controls. The hearing thresholds were significantly higher in patients with presbycusis than controls (*p < 0.001; 1,000–8,000 Hz). Data are presented as mean ± SD.

Notably, 51 patients with presbycusis and 60 HCs were included in the final analysis. The clinical and neuropsychological characteristics of both groups are summarized in Tables 1, 2. There were no significant differences between the presbycusis group and HCs in terms of age, sex, education level, or middle-ear function (because all participants had a type A curve on tympanometry, which indicates normal function). In addition, no significant differences were found in the PTA between the left and right ears of the patients with presbycusis and the HCs. A summary of the average hearing thresholds of both ears in all subjects is presented in Figure 1. The average PTA of patients with presbycusis was significantly higher than that of the HCs (*p* < 0.001, 1,000–8,000 Hz). In terms of the cognitive assessment, patients with presbycusis performed significantly worse, with lower DST and TMT-B scores (*p* < 0.05). Significant differences in the other cognitive assessments were not detected.

**TABLE 1:** Demographics of patients with presbycusis and the healthy controls (HCs).

	Patients with presbycusis (*n* = 51)	Healthy controls (*n* = 60)	*p*-value
Age (year)	61.65 ± 8.24	59.78 ± 5.15	0.165
Gender (male: female)	24/27	27/33	0.828
Education (years)	10.80 ± 2.03	10.80 ± 1.70	0.991
PTA of left ear (dB HL)	33.02 ± 4.06	15.88 ± 2.79	<0.001*
PTA of right ear (dB HL)	32.65 ± 6.29	16.22 ± 3.11	<0.001*
Mean PTA of both ears (dB HL)	32.84 ± 3.85	16.05 ± 2.16	<0.001*

Data are represented as mean ± SD. *p < 0.001. PTA, pure tone audiometry.

**TABLE 2:** Neuropsychological scores of patients with presbycusis and HCs.

	Patients with presbycusis (*n* = 51)	Healthy controls (*n* = 60)	*p*-value
MMSE	28.96 ± 1.26	28.85 ± 1.36	0.666
MoCA	25.84 ± 1.77	26.46 ± 1.80	0.072
AVLT	33.49 ± 7.24	34.93 ± 6.94	0.285
CFT	34.50 ± 1.71	34.43 ± 1.71	0.816
CFT-delay	16.68 ± 3.71	17.21 ± 4.25	0.483
TMT-A	69.08 ± 20.37	70.56 ± 20.71	0.705
TMT-B	171.35 ± 51.49	151.03 ± 45.58	0.031*
CDT	3.47 ± 0.54	3.54 ± 0.53	0.492
DST	11.20 ± 1.58	11.80 ± 1.90	0.067
VFT	14.71 ± 4.05	14.91 ± 3.62	0.779
DSST	69.90 ± 8.43	68.85 ± 9.36	0.538
SAS	36.67 ± 5.86	35.66 ± 6.51	0.394
SDS	38.49 ± 8.64	36.34 ± 8.02	0.176

Data are represented as mean ± SD, *p < 0.05. MMSE, Mini-Mental State Examination; MoCA, Montreal Cognitive Assessment; AVLT, Auditory Verbal Learning Test; CFT, Complex Figure Test; DST, Digit Span Test; TMT-A, Trail Making Test-Part A; TMT-B, Trail Making Test-Part B; CDT, Clock Drawing Test; VFT, Verbal Fluency Test; DSST, Digit Symbol Substitution Test; SDS, Self-Rating Depression Scale; SAS, Self-Rating Anxiety Scale.

### Resting-State Network Alterations

**FIGURE 2:**
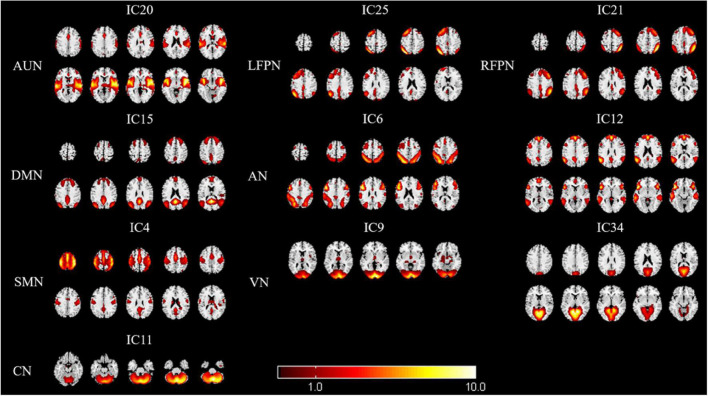
Spatial maps of identified resting-state networks (RSNs) are divided into eight different functional domains, namely, AUN, LFPN, RFPN, DMN, AN, SMN, VN, and CN. AUN, auditory network; LFPN, left frontoparietal network; RFPN, right frontoparietal network; DMN, default mode network; AN, attention network; SMN, sensorimotor network; VN, visual network; and CN, cerebellum network.

We identified eight RSNs (10 ICs) from the fMRI data after the ICA, with spatial distributions similar to those of previous research. The AUN (IC20) was composed of the superior temporal gyrus and MTG, which are responsible for auditory processing. The DMN (IC15) comprised the medial prefrontal cortex, posterior cingulate cortex/precuneus, bilateral inferior parietal lobe, and angular gyrus. The AN (IC6 + 12) included the dorsal domain and the ventral domain, which mainly encompassed the bilateral intraparietal sulcus, frontal eye field, temporoparietal junction area, and ventral frontal cortex. The LFPN (IC25) and RFPN (IC21) were concentrated in the prefrontal cortex and the posterior parietal cortex. The SMN (IC4) mainly included the precentral gyrus and part of the postcentral gyrus. The VN (IC9 + 34) and CN (IC11) encompassed the occipital pole and cerebellar cortex, respectively, which are consistent with prior anatomical and functional delineations (Figure 2).

### Static Functional Network Connectivity and Dynamic Functional Network Connectivity

**FIGURE 3:**
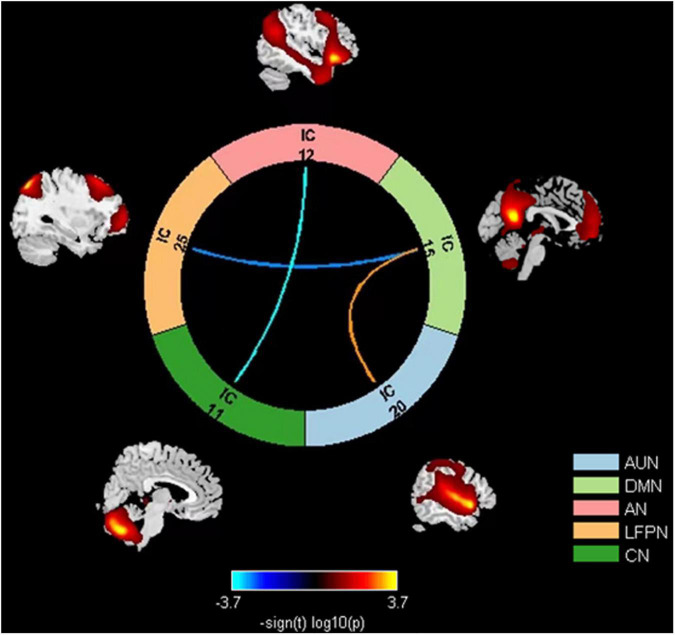
Group comparison results of sFNC between eight RSNs: decreased sFNC in the DMN (IC15)-LFPN (IC25) and AN (IC12)-CN (IC11) pairs and increased sFNC in the AUN (IC20)-DMN (IC15). sFNC, static functional network connectivity; DMN, default mode network; LFPN, left frontoparietal network; AN, attention network; CN, cerebellum network; AUN, auditory network.

The results of the sFNC analysis for patients with presbycusis and HCs are shown in Figure 3. Significant differences were found in network connectivity in the AUN, AN, DMN, LFPN, and CN between these two groups. Compared with HCs, patients with presbycusis had decreased sFNC between DMN (IC15) and LFPN (IC25), as well as between AN (IC12) and CN (IC11). The presbycusis group exhibited significantly increased sFNC in the AUN (20)-DMN (15) pairs.

**FIGURE 4:**
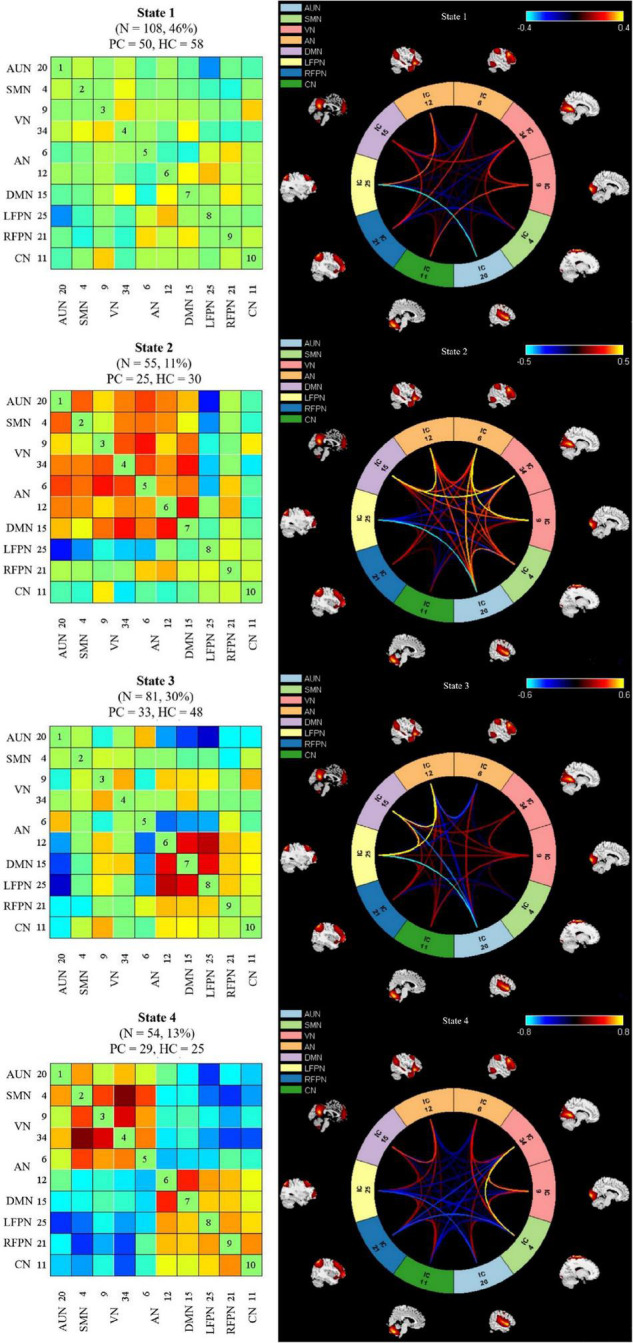
Left column indicates dynamic functional network connectivity (dFNC) centroids of the four states, the number of subjects, and the percentage of occurrence in each state. Right column shows the visualization of dFNC in each state. PC, presbycusis; HC, healthy controls; AUN, auditory network; SMN, sensorimotor network; VN, visual network; LFPN, left frontoparietal network; RFPN, right frontoparietal network; DMN, default mode network; AN, attention network; CN, cerebellum network.

Time-varying FNC during scanning was clustered into four states by k-means clustering; the centroids of the four states are presented in Figure 4. State 1 accounted for 46% of all windows and had the largest occurrence frequency. It is worth noting that the total number of subjects per state varied, as not all subjects entered into all states. The majority of the subjects (108/111) experienced State 1 and State 3 (81/111), which had the highest occurrence rates. In addition, 55 subjects experienced State 2, and 54 experienced State 4. The number of presbycusis and HC subjects in each state was similar, indicating that the two groups were equally represented across states despite the reduced number of subjects. State 1 mostly exhibited weak connectivity among all networks. That is, the connections were mainly sparse. State 2 accounted for 11% of all windows; in this state, there were highly positive FNCs within and between all RSNs, except for the relatively weaker FNCs between FPN and CN related to the other networks. State 3 was characterized by highly positive connections between AN, DMN, and LFPN. State 4 was similar to State 3, with strong positive connectivity between SMN, VN, and AN; the AN and DMN were highly connected, while the other RSNs had relatively weaker or negative FNCs in States 3 and 4.

**FIGURE 5:**
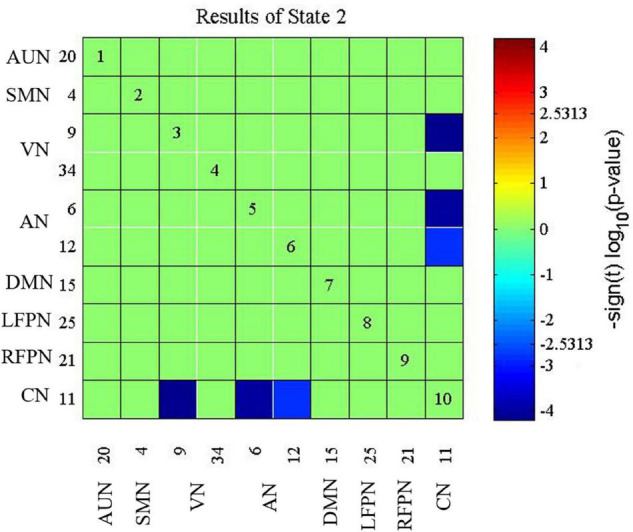
Connectivity results for State 2 evaluated using two-sample *t*-tests, and the significance was corrected using false discovery rate (FDR): decreased dynamic functional network connectivity (dFNC) in the CN (IC11) with AN (IC6+12) and VN (IC9). CN, cerebellum network; AN, attention network; VN, visual network.

The presbycusis group exhibited an abnormal decrease in transient dFNC patterns compared to HCs in State 2, which are illustrated in Figure 5. In State 2, significantly decreased dFNCs were found between CN (IC11), AN (IC6+12), and VN (IC9). Moreover, patients with presbycusis demonstrated significantly shorter MDT and FT in State 3 than HCs (*p* = 0.0027 and *p* = 0.0031, respectively) (Tables 3, 4). However, no significant difference in the NT of states between presbycusis group and HCs was found (*p* > 0.05).

**TABLE 3:** Two sample *t*-test of mean dwell time between the patients with presbycusis and the HCs.

State	Presbycusis (*n* = 51)	Healthy controls (*n* = 60)	*p*-value	*t*-value
1	56.1582 ± 55.6921	37.9664 ± 41.1299	0.0529	1.9569
2	16.6275 ± 25.7619	10.3917 ± 16.5388	0.1302	1.5247
3	16.1134 ± 18.5945	30.6181 ± 28.7204	0.0027*	–3.0689*
4	15.3072 ± 18.0488	10.9686 ± 16.5557	0.1936	1.3080

Data are the mean ± SD; *indicates significant difference.

**TABLE 4:** Two sample *t*-test of fraction time between the patients with presbycusis and the HCs.

State	Presbycusis (*n* = 51)	Healthy controls (*n* = 60)	*p*-value	*t*-value
1	0.5192 ± 0.3149	0.4112 ± 0.2641	0.0541	1.9467
2	0.1331 ± 0.2072	0.0907 ± 0.1537	0.2234	1.2246
3	0.2076 ± 0.2532	0.3696 ± 0.2982	0.0031*	–3.0279*
4	0.1400 ± 0.1893	0.1284 ± 0.2330	0.7781	0.2825

Data are the mean ± SD; *indicates significant difference.

### Correlation Results

The correlations between FC attributes and cognitive performance in the presbycusis group were further analyzed. A significant negative correlation was found between the sFNC differences in the DMN-LFPN pair and the TMT-B score (*r* = –0.441, *p* = 0.002). However, the other sFNC or dFNC differences were not significantly correlated with cognitive performance. Moreover, no significant correlations among the dFNC indices (i.e., MDT, FT, and NT) and cognitive scores were observed in patients with presbycusis.

## Discussion

This was the first study to combine sFNC analysis with dFNC analyses; thus, compared to the previous purely static evaluations, the brain dynamics were fully considered. Studies have shown abnormal network interactions in the DMN, LFPN, AN, CN, and VN in patients with presbycusis. The current results support the hypothesis that abnormal static and dynamic connectivity patterns in rs-FC are associated with cognitive impairment in patients with presbycusis, emphasizing the importance of investigating rs-FC from both static and dynamic perspectives as that can provide additional relevant information on the disease and help to map the full picture of connectivity abnormalities.

The DMN, i.e., the key brain network in the resting state, remains deactivated during tasks requiring external attention and is mainly responsible for social cognition, working memory, decision-making, and awareness (Buckner and DiNicola, 2019). Previous studies have discovered that patients with presbycusis have significantly reduced FC in the DMN and that there are abnormal interactions between DMN and other networks (Xu et al., 2019b; Zhang and Volkow, 2019). In addition, the neural consequences of hearing loss include structural alterations of the cortex, impacting cognitive function, and gray matter atrophy, including the bilateral precuneus, cingulate cortex, and insula; a thicker insula is related to better speech perception (Ren et al., 2018). The LFPN can actually be regarded as a language network, and its left lateralization is consistent with the laterality of language areas, which govern language-related cognition (Zhu et al., 2014). Disrupted FC between FPN and DMN was observed in schizophrenia and healthy subjects with sleep deprivation (Stolzberg et al., 2018), suggesting that sFNC may serve as a biomarker of impaired function. It is well known that hearing loss leads to reduced central auditory activation and that the changes in the AUN negatively impact auditory perception and verbal communication ability. Our results showed increased sFNC between DMN and AUN and decreased sFNC between DMN and LFPN, similar to those of previous studies. This finding supports the concept of auditory cortex plasticity and, further, may represent functional compensation, which suggests that patients with presbycusis tend to recruit more cognitive resources to support auditory perception, leading to the impairment of higher-order cognitive behaviors such as semantic perception and goal orientation.

An increasing number of studies have confirmed that the CN plays an indispensable role in hearing, mainly reflected by the fact that the CN is second only to the primary auditory cortex in auditory processing and is the most active brain area for hearing-related tasks (Xu et al., 2019a; Shahsavarani et al., 2021). Neuroimaging studies have also discovered the activation of the cerebellum during auditory input, and auditory deprivation has been found to interfere with communication between CN and other cortical networks (Brady et al., 2019). In addition, hearing-impaired patients experience increased recruitment of the CN after hearing aid use (Vogelzang et al., 2021). Cerebellar output is anatomically and functionally connected to the frontal cortex through the subcortical area, forming a cerebrocerebellar circuit. The AN involves the frontal cortex, including the frontal eye field and the ventral frontal cortex. We observed a reduction in the sFNC and dFNC between CN and AN, suggesting that due to the decrease in peripheral auditory input, the activation of the CN was reduced, and the cerebrocerebellar circuit connection was interrupted accordingly. The decline in attention and the attention-related cortical atrophy in patients with hearing loss (Fortunato et al., 2016) are similar to our results, reflecting impaired AN function, as the AN participates in the perceptual analysis and processing of auditory signals. The perceptual understanding of auditory information requires integration among brain networks (Slade et al., 2020), which further leads to difficulty in understanding speech in patients with presbycusis. Thus, patients strive to allocate more neural resources to listening, and correspondingly, higher-level cognitive resources decrease, thereby confirming that the mechanism of the relationship between presbycusis and cognitive impairment is the reallocation of cognitive resources.

It is worth noting that although the cerebellum was initially considered to mainly control motion, later studies have verified its important role in multimodal integration (Xiao and Scheiffele, 2018). The cerebellum contains afferent fibers of the visual sensory system, conveying a wealth of visual information and directing visual attention. Our study observed that patients with presbycusis showed reduced dFNC between the VN and the CN, suggesting abnormalities in visual-motor integration, which is consistent with the previous findings of decreased FC in the motor area of patients with hearing loss (Chen et al., 2020). Studies have also suggested that the decreased connectivity between auditory and motor areas is related to stronger audio-visual integration (Schulte et al., 2020). Our results may therefore indicate that the partial deprivation of auditory perception affects motor function in much the same way that the partial deprivation of one sensory modality influences the function of the rest of the sensory modalities (Schmithorst et al., 2014). Several longitudinal studies (Kamil et al., 2016; Jayakody et al., 2018) have shown an increased risk of falls in older patients with hearing loss, which may be caused either by hearing loss, which reduces the ability to locomote and balance and thus leads to falls, or by cochlear dysfunction, which leads to impairments in spatial and directional hearing and acoustic orientation. As is currently well known, the role of the cerebellum has shifted from one that is purely sensorimotor related to one that involves a broad range of cognitive functions. Our results not only provide a reasonable explanation for falls but also suggest the neural mechanism of cognitive impairment in patients with presbycusis.

We found that patients with presbycusis have aberrant dFNC temporal properties. State 1, which has sparse connectivity, was the most frequent brain state, followed by States 3 and 4. Although State 2 was the least frequent brain state, it mainly presented as highly positive connectivity, reflecting interconnection between brain networks. The dFNC patterns vary among different states, suggesting flexibility in functional coordination between brain networks. State 3 was characterized by a close relationship between AN, DMN, and LFPN. Patients with presbycusis spent the shortest FT and MDT in State 3, but this short stay may not be conducive to a functional interaction between the aforementioned networks, therefore presenting a mechanism of cognitive impairment. We also found that patients with presbycusis tended to remain in a state with sparse FC (State 1), although this difference was not significant when compared to the control group. Sparse FC usually means inefficient functional integration, which is a characteristic that is adverse to the fluency of brain cognitive resource allocation. Therefore, we speculated that it might be the reason for its cognitive deficits.

This study had several limitations. First, it is relatively limited in inferring causal associations from the observed results. Future studies should use a longitudinal design to evaluate the sensitivity and specificity of FNC analysis in cognitive impairment in presbycusis. Second, the functional networks involved in the research were based on multiple components determined by ICA; other networks, such as the salience network and basal ganglia network, which may have important impacts on the neural mechanisms of presbycusis, were not taken into account; thus, the follow-up research should include a more comprehensive network interaction model. Third, other methods, such as graph theoretical approaches (Jia et al., 2020), using time-frequency information (Yaesoubi et al., 2015) or coactivation patterns (Bolton et al., 2020), also show promise in identifying information that static methods fail to capture. Finally, although the subjects were told not to think about anything in particular and to keep relatively still during the scanning process, we still could not identify what subjects actually thought about. Further research should explore ways to work around these limitations and further clarify the dynamic FC changes in patients with presbycusis.

## Conclusion

In summary, this study revealed abnormal sFNC and dFNC and altered temporal properties of dynamic FC in patients with presbycusis and found that they were correlated with neurocognitive changes. These findings enrich our understanding of the neural mechanisms underlying cognitive impairment associated with presbycusis and may serve as a potential imaging biomarker for investigating and predicting cognitive difficulties. Furthermore, the current findings might contribute to earlier clinical diagnosis, prevention, and treatment of presbycusis on account of brain connectomics.

## Data Availability Statement

The original contributions presented in the study are included in the article/supplementary material, further inquiries can be directed to the corresponding author/s.

## Ethics Statement

The studies involving human participants were reviewed and approved by the Research Ethics Committee of Nanjing Medical University. The patients/participants provided their written informed consent to participate in this study.

## Author Contributions

CX and Y-CC designed the experiment, analyzed the data, and drafted the manuscript for the work. S’aS, J-JX, and HC helped to acquire the clinical and fMRI data. XY helped to revise the manuscript critically for important intellectual content. YW and J-XZ did the financial support, review, and final approval of the manuscript to be published. All authors have read and approved the final manuscript.

## Conflict of Interest

The authors declare that the research was conducted in the absence of any commercial or financial relationships that could be construed as a potential conflict of interest.

## Publisher’s Note

All claims expressed in this article are solely those of the authors and do not necessarily represent those of their affiliated organizations, or those of the publisher, the editors and the reviewers. Any product that may be evaluated in this article, or claim that may be made by its manufacturer, is not guaranteed or endorsed by the publisher.
